# Case Report: Epileptic phenotype in a patient with a *MARK2* variant: the first detailed description and review of the literature

**DOI:** 10.3389/fped.2026.1768748

**Published:** 2026-05-21

**Authors:** Jin Sook Lee, You Min Kang, Yeseul Kim, Young Ok Kim, Jong Hee Chae

**Affiliations:** 1 Department of Pediatrics, Seoul National University Children’s Hospital, Seoul, Republic of Korea; 2 Department of Pediatrics, Chonnam National University Children’s Hospital, Gwangju, Republic of Korea; 3 Department of Pediatrics, Chonnam National University Medical School, Gwangju, Republic of Korea; 4 Department of Laboratory Medicine, Seoul National University Hospital, Seoul National University College of Medicine, Seoul, Republic of Korea; 5 Department of Pediatrics, Seoul National University College of Medicine, Seoul, Republic of Korea; 6 Department of Genomic Medicine, Seoul National University Hospital, Seoul, Republic of Korea

**Keywords:** child, exome sequencing, genes, intellectual disability, seizures, therapeutics

## Abstract

**Background:**

The gene encoding microtubule affinity-regulating kinase 2 (*MARK2*) has recently been implicated in patients with autism spectrum disorder (ASD). Although seizures have been reported in 46.4% of a small cohort, detailed information regarding seizure phenotypes and longitudinal progression remains scarce. We present a case of *MARK2*-related epilepsy and review previously reported cases in the literature.

**Case report and review:**

An 11-year-old male patient with ASD carrying a heterozygous pathogenic variant (c.888 + 1G > A) in *MARK2* gene experienced his first nocturnal tonic seizure at 5 years 11 months. Recurrent nocturnal focal impaired-consciousness seizures were accompanied by left temporal sharp waves on EEG, and episodes of focal status epilepticus were also noted. Oxcarbazepine provided only partial benefit, whereas perampanel effectively reduced nocturnal seizures and improved sleep initiation. He has remained seizure-free since 9 years 9 months on combined therapy. In our literature review, no prior reports described longitudinal changes in seizure manifestations, EEG evolution, or treatment response. EEG abnormalities were noted in 72.7% of patients (8/11), with focal epileptiform discharges being the most common finding (*N* = 5). No major structural abnormalities were identified on brain MRI.

**Conclusion:**

This case provides the first detailed clinical description of *MARK2*-related epilepsy, characterized by focal seizures responsive to antiseizure medication.

## 1 Introduction

Epilepsy is not uncommon among children with neurodevelopmental disorders (1). Both conditions are believed to have genetic etiologies, many of which have been rapidly identified with the advent of next-generation sequencing (2–4). The genes implicated in these disorders help explain why patients with neurodevelopmental conditions often experience additional seizures—they can be understood as divergent clinical manifestations originating from a shared genetic basis (2, 5). Epilepsy patients carrying gene variants within the same gene that lead to similar functional impairments often exhibit similar patterns of seizure progression and treatment responses (6). Therefore, delineating the phenotypic spectrum associated with pathogenic variants in a specific gene is clinically meaningful for physicians caring for these patients.

The gene encoding microtubule affinity–regulating kinase 2, *MARK2* (MIM:600526), has been associated with intellectual developmental disorder, autosomal dominant 76 (MIM:621285) and has recently gained attention after being identified as a novel risk gene in patients with autism spectrum disorder (ASD) (4). *MARK2* plays an essential role in neuronal polarity and contributes to dendritic development (7, 8). Pathogenic variants in *MARK2* are considered to confer a moderate risk for ASD, likely through mixed or non-canonical pathogenic mechanisms rather than a single archetypal pathway (4). In Gong et al.'s cohort, seizures were reported in 46.4% of patients (13 of 28 with available clinical information); however, detailed seizure phenotypes were not provided (9). Sun et al. described a single case with seizures, including information on the initial seizure event, electroencephalography (EEG), and brain magnetic resonance imaging (MRI) findings, but did not report subsequent seizure evolution, antiseizure medication choices, or treatment responses (10).

Here, we describe a case with a pathogenic *de novo MARK2* variant, including detailed longitudinal information on seizure manifestations, EEG evolution, and therapeutic response. In addition, we review and summarize the clinical features of the fifteen previously reported individuals with pathogenic or likely pathogenic *MARK2* variants who experienced seizures, incorporating our current case.

## 2 Case report

### 2.1 Case description in our proband

#### 2.1.1 Developmental and Clinical Background

The male patient was delivered at 38 weeks of gestation via cesarean section due to advanced maternal age, with a birth weight of 3 kg. His family history was notable for a paternal uncle with intellectual disability, while both parents were healthy and non-consanguineous. Developmental delay was apparent, as independent walking was achieved at 18 months and eye contact remained poor. No structural anomalies or facial dysmorphic features were identified. Brain MRI performed at 3 years and 7 months revealed no abnormal findings. At 4 years, the Childhood Autism Rating Scale (CARS) score suggested mild to moderate autism (score 31), and the Social Maturity Scale (SMS) estimated a social age equivalent to 2 years and 2 months. The Sequenced Language Scale for Infants (SELSI) demonstrated receptive language skills at a 4-month level and expressive language skills at a 7-month level. A diagnosis of autism spectrum disorder was subsequently made by a child psychiatrist (Table 1).

**Table 1:** Characteristics of patients with *MARK2* gene^a^ and seizures.

Clinical category		Proband	Sun et al. (10)	Gong et al. (9)	Total N (%)
Number		1	1	13	15 (100.0)
Gender (male)	Male	1	1	8	10 (66.7)
Age at last visit	Early childhood (3–5 years)	0	1	5	6 (40.0)
	Late childhood (6–9 years)	0	0	5	5 (33.3)
	Adolescence (10–17 years)	1	0	1	2 (16.7)
	Adulthood (>18 years)	0	0	2	2 (16.7)
Diagnostic method	WES	1	1	10	12 (80.0)
	WGS	0	0	3	3 (20.0)
Genomic position	Exon	0	1	11	12 (80.0)
	Intron	1	0	2	3 (20.0)
Mutation category-	Stop	0	1	4	5 (33.3)
	Frameshift	0	0	5	5 (33.3)
	Missense	0	0	2	2 (13.3)
	Splice	1	0	2	3 (20.0)
Inheritance (Total *N* = 11)	*De novo*	1	1	8	10 (90.1))
	Autosomal dominant	0	0	1	1 (9.0)
Pathogenicity	Pathogenic	1	1	7	9 (60.0)
	Likely pathogenic	0	0	6	6 (40.0)
Seizure semiology	Focal seizures	1	1	NA	2 (100.0)
(Total *N* = 2)	Generalized seizures	0	NA	NA	
Seizure onset age (Total *N* = 2)	Early childhood (3–5 years)	1	1	0	2 (100.0)
EEG (Total *N* = 11)	Generalized ED	0	0	2 (1)^b^	2^b^ (18.2)
	Focal ED	1	1	3 (1)^b^	5^b^ (45.5)
	Abnormal without SI	0	0	2	2 (18.2)
	Normal	0	0	3	3 (27.3)
Brain MRI (Total *N* = 11)	Minor abnormalities	0	0	4	4 (36.4)
	Normal	1	1	5	7 (63.6)
ASD	(Total *N* = 15)	1	1	12	14 (93.3)
Intellectual disability	(Total *N* = 14)	1	1	12	14 (100.0)
Motor delay	(Total *N* = 14)	1	0	7	8 (57.1)
Hypotonia/hypertonia	(Total *N* = 12)	0	0	1	1 (8.3)
Language regression^c^	(Total *N* = 12)	0	NA	4	4 (33.3)
Sleep problems	(Total *N* = 14)	1	NA	3	4 (28.6)
Behavioral problems^d^	(Total *N* = 13)	0	NA	9	9 (69.2)
Facial dysmorphism	(Total *N* = 10)	0	0	7	7 (70.0)
CHD	(Total *N* = 12)	0	0	1	1 (8.3)

N, number; WES, whole-exome sequencing; WGS, whole-genome sequencing; EEG, electroencephalography; ED, epileptiform discharge; SI, specific information; ASD, autism spectrum disorder; NA, not available; CHD, congenital heart defect.

a All variants of *MARK2* were heterozygous.

b One patient exhibited both generalized and focal epileptiform discharges.

c Language problems were reported in all 15 patients.

d Behavioral problems included attention deficit/hyperactivity disorder, depression, aggression and anxiety.

At 6 years and 5 months, his body weight was 17.7 kg (3rd–5th percentile), height 118 cm (50th–75th percentile), and head circumference 53 cm (75th–90th percentile). However, growth gradually slowed, and at 10 years and 6 months, his body weight was 25 kg (<3rd percentile) and height 132 cm (3rd–5th percentile).

#### 2.1.2 Seizures and EEGs

**Figure 1:**
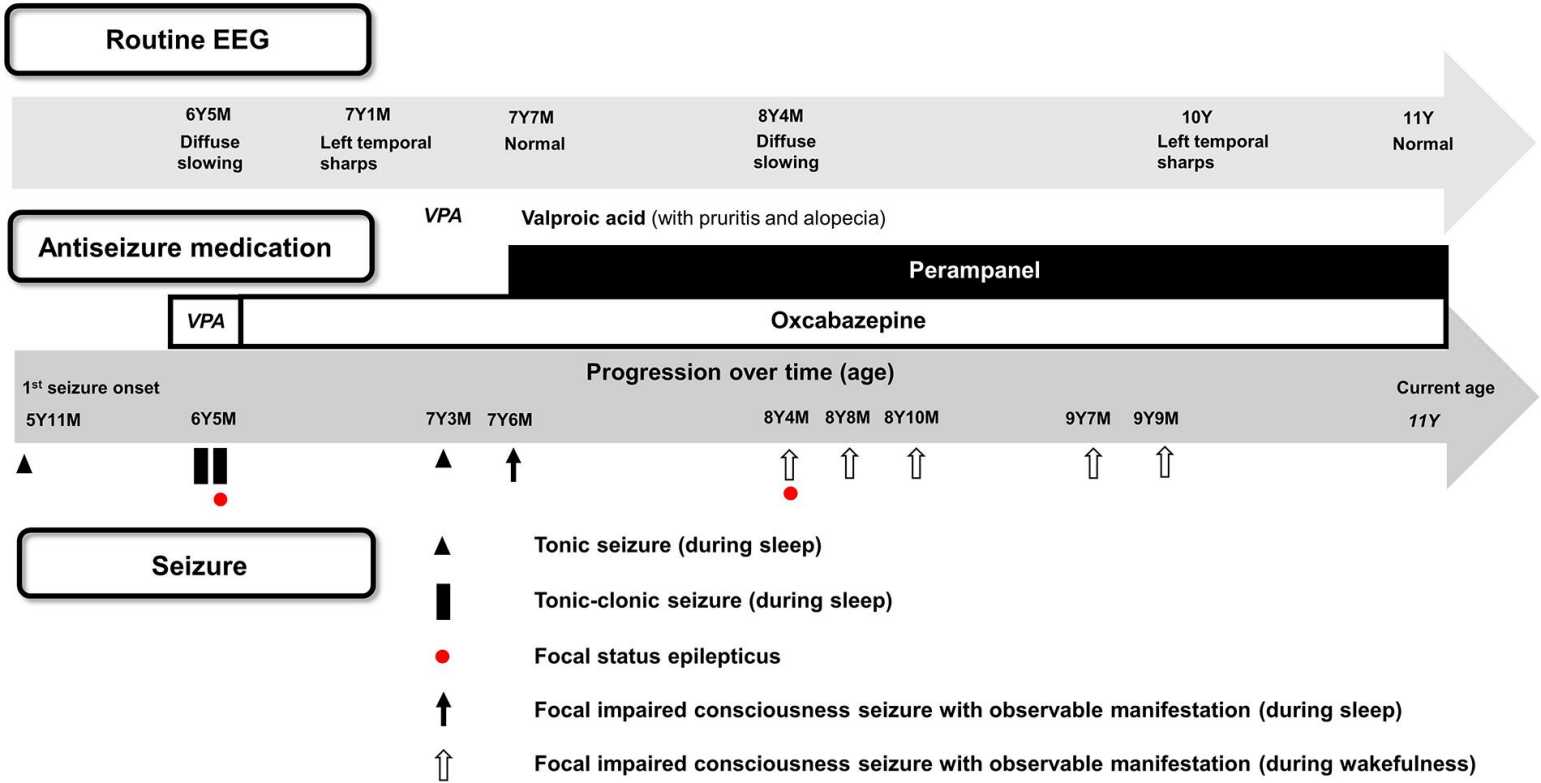
Seizure progression, EEG findings, and treatment response. Most seizures, first occurring at 5 years and 11 months of age, were focal in nature and associated with intermittent focal epileptiform discharges on EEG. They were effectively controlled with oxcarbazepine and perampanel. No nocturnal focal seizures were observed after the initiation of perampanel.

**Figure 2:**
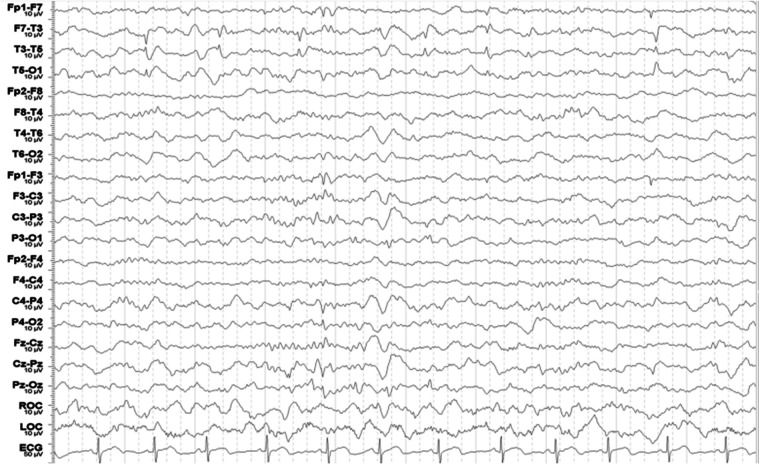
Sleep EEG at 7 years and 1 month of age, demonstrating left temporal sharp waves with maximal negativity at T3.

At 5 year and 11 months, he experienced his first tonic seizure during sleep, characterized by clenching his hands, perioral cyanosis, and a fixed gaze toward one side for 5–10 min. Although he was brought to our emergency department, he subsequently discontinued outpatient follow-up. At 6 years and 5 months of age, he presented the second nocturnal tonic-conic (TC) seizure lasting 2 min. In sleep EEG, only postictal diffuse slowing was noted. Two weeks later, another similar nocturnal TC seizure lasting for 3–4 min occurred and then resulted in persistent impaired consciousness with eyeball deviation, consistent with focal status epilepticus (SE), which was ameliorated by intravenous lorazepam. Initially, valproic acid was prescribed, but was discontinued due to pruritus and alopecia. Considering the possibility of focal epilepsy, oxcarbazepine (OXCBZ) was started. Follow-up EEG at 7 years and 1 month of age demonstrated the left temporal sharp waves (Figures 1, 2).

At 7 years and 3 months, a brief nocturnal tonic seizure occurred. Three months later, another nocturnal focal impaired consciousness seizure occurred: the patient awoke with an unusual sensation similar to his prior seizure onset, uttered a short “ah” sound, and exhibited brief clonic jerks in upper extremities. Perampanel (PER) was added. At 8 years and 4 months, focal SE occurred during a febrile illness: a seizure began with eye deviation, and then bilateral TC movements. It persisted for 25 min despite intravenous lorazepam. At 8 years and 8 months and two months later, short focal impaired consciousness seizures occurred again, characterized by an uncomfortable facial expression, and loss of postural control. At 9 years and 9 months, a focal impaired consciousness seizure reappeared: after vomiting the patient became cyanotic and apneic, with eye deviation. Sleep EEG showed intermittent left temporal sharp waves at T3 again. Although irritability increased after PER dose adjustments, nocturnal seizures resolved, and sleep initiation improved. Since then, no seizures have recurred, and the patient is currently 11 years old (Figure 1).

#### 2.1.3 Genetic diagnosis

Chromosome analysis and chromosomal microarray performed at 3 years and 8 months were normal, as was the fragile X test. Trio whole-exome sequencing after 9 years of age revealed a heterozygous pathogenic variant (c.888 + 1G > A) in *MARK2* gene (NM_001039469.3). This rare canonical splice-site variant was *de novo*, and was not found in normal population database of human (e.g., ExAC or gnomAD).

### 2.2 Review of fifteen *MARK2* variant cases with reported seizures and documented clinical features

There have been 15 reported cases of *MARK2* variants with seizures and documented clinical features in the literature, including our own (9, 10). Seizure phenotypes and age at onset were described in only two patients—our case and the case reported by Sun et al.—both of whom presented with nocturnal focal impaired consciousness seizures with observable manifestation beginning in early childhood (around 5 years of age) (10). EEG abnormalities were observed in 72.7% of 11 patients with available data. Focal epileptiform discharges—including one patient who exhibited both generalized and focal discharges—were the most frequent finding (*N* = 5). Brain MRI findings were normal in 63.6% of patients, with only minor abnormalities reported in the remaining 36.4% (of 11 total patients). Variants were classified as stop and frameshift (*N* = 5 each, 33.3%), splice-site (*N* = 3, 20.0%), and missense variants (*N* = 2, 13.3%). No mutational hotspot was identified. Among 11 patients with available segregation data, 90.1% of variants were *de novo* (Table 1).

ASD was reported in 93.3% of patients, and intellectual disability was present in all 14 patients assessed. Motor delay was noted in 57.1% of 14 patients. Language regression was observed in 33.3% of 12 patients, although language problems were reported in all 15 patients. None of the 14 patients with available data had hearing impairment. Sleep and behavioral problems were reported in 28.6% and 69.2% of patients, respectively. Facial dysmorphism was identified in 70.0% of patients.

## 3 Discussion

The present report provides the first detailed clinical characterization of *MARK2*-related epilepsy, including longitudinal information on seizure semiology, EEG evolution, and treatment response. Our proband, who has epilepsy and ASD and carries a heterozygous pathogenic variant in the *MARK2* gene (c.888 + 1G > A), experienced his first nocturnal tonic seizure at 5 years 11 months of age. He subsequently developed recurrent nocturnal focal impaired-consciousness seizures, accompanied by left temporal sharp waves on EEG, and episodes of focal SE were also observed. His seizures were controlled with OXC and PER, with PER demonstrating notable efficacy in reducing nocturnal seizures and sleep initiation. To date, seizure phenotypes and age at onset have been described in only two patients, including our proband, both of whom exhibited focal impaired-consciousness seizures arising during sleep in early childhood (around 5 years of age) (10). EEG findings, available for 11 patients, were abnormal in 72.7%, with focal epileptiform discharges being the most common abnormality (*N* = 5). No major structural abnormalities were identified on brain MRI (9, 10). This case report delineates the phenotypic spectrum of *MARK2*-related epilepsy, and our review of the literature highlights the need for further studies to clarify and expand this emerging epileptic phenotype.

The case reported by Sun et al. was described as having a mild phenotype, presenting with ASD, epilepsy, and developmental delay, but without major anomalies or dysmorphic features (10). His first seizure, at 5 years 3 months of age, occurred during morning sleep. It manifested as focal status epilepticus that began with sudden eye opening, eyeball deviation, and drooling, and lasted for approximately two hours until relieved by intravenous midazolam. EEG showed focal sharp/spike-and-slow-wave discharges in the right occipital and temporal regions. He was presumed to have a favorable seizure outcome, as he experienced only one additional seizure after the initial event and remained on antiseizure medication without further recurrence; however, the specific medication used and the duration of follow-up were not reported. The case described by Sun et al. shares notable similarities with our own case (10).

Clinical phenotypes associated with *MARK2* variants were first described in 2024 (9). In a small cohort comprising 31 patients with *MARK2* variants, intellectual disability/developmental delay and speech/language problems were reported in all patients with available data (29 and 31 patients, respectively). Behavioral problems, including ADHD, aggression, anxiety, and depression, were noted in 74.1% of 27 patients. Motor delay (62.1% of 29 patients), language regression (40.9% of 22 patients), ophthalmologic problems (39.3% of 28 patients), and genital malformations in males (33.3% of 18 patients) were also reported. Distinct facial features were commonly observed, including a narrow face, broad and prominent forehead, down-slanted palpebral fissures, a broad nasal root, and large dysmorphic ears (9). In our review of patients with seizures and *MARK2* variants, sleep problems such as insomnia were identified in 28.6% of 14 patients (9, 10). Overall, the clinical characteristics of patients with seizures were largely consistent with those previously reported for *MARK2*-related disorders.

In conclusion, our case report provides the first detailed clinical description of *MARK2*-related epilepsy, characterized by focal seizures responsive to antiseizure medication. This case delineates the phenotypic spectrum of *MARK2*-related epilepsy, and our review of the literature underscores the need for further studies to clarify and expand this emerging epileptic phenotype. Moreover, efforts are warranted to identify clinical biomarkers that can distinguish patients with seizures from those without, thereby improving prediction of seizure susceptibility in individuals with *MARK2* variants.

## Data Availability

The original contributions presented in the study are included in the article/supplementary material, further inquiries can be directed to the corresponding author/s.
